# Heat fluxes and thermal stability in the mixolimnion of a tropical heliothermal meromictic crater lake

**DOI:** 10.1038/s41598-025-25195-x

**Published:** 2025-11-21

**Authors:** E. Palacios-Hernández, A. Filonov, D. Avalos-Cueva, L. Carrillo, Cesar O. Monzón

**Affiliations:** 1 Physics Department, University of Guadalajara, 1500 Revolución, Olímpica Sector, Guadalajara, Jalisco México; 2 Department of Civil Engineering and Topography, University of Guadalajara, 1421 Blvd. Marcelino García Barragán, Guadalajara, 44430 Mexico; 3 El Colegio de la Frontera Sur, Departamento de Observación y Estudios de la Tierra, la Atmósfera y el Océano, Av. Centenario km 5.5, Col. Pacto Obrero, 77014 Chetumal, Quintana Roo Mexico; 4 Department of Project Engineering, University of Guadalajara, Blvd. Jose Guadalupe Zuno 48, Industrial los Belenes, Zapopan, 45157 Jalisco Mexico

**Keywords:** Meromictic, Heliotermal lake, Heat fluxes, Vertical mixing, Tropical limnology, Schmidt stability index, Climate sciences, Ocean sciences

## Abstract

An 11-month high-resolution dataset from a rare, hypersaline crater lake on Mexico’s Isla Isabel reveals its intense heliothermal regime is not a permanent state but a distinct seasonal phenomenon. The development of a subsurface temperature maximum exceeding 47 °C is driven by ectogenic meromixis, where a massive influx of clear freshwater during the rainy season forms a low-density surface cap, suppressing vertical mixing and trapping solar radiation in the denser, saltier layers below. Salinity is therefore the fundamental control on the lake’s physical dynamics, creating the precondition for massive subsurface energy storage. This study provides the first definitive characterization of a complete annual cycle in a tropical heliothermal lake, establishing it as a critical model system for understanding the physical stability and response of these rare ecosystems to seasonal climatic forcing.

## Introduction

Lakes are exceptional sentinels of regional climate, responding with high fidelity to seasonal and inter-annual shifts in atmospheric forcing such as temperature, precipitation, and wind dynamics^1–5^. Among the diverse array of lacustrine environments, tropical meromictic lakes are of particular scientific interest. Their permanent, salinity-driven stratification makes them highly stable systems, yet their surface layers (the mixolimnion) can exhibit extreme responses to seasonal forcing. This offers a unique natural laboratory to study heat transfer and stability processes under high-irradiance conditions. Despite their importance, the physical dynamics governing seasonal heat storage in these systems remain poorly quantified compared to their well-studied temperate counterparts, representing a significant gap in our understanding of lacustrine physics.

The primary source of energy for a lake’s heat budget is solar radiation, which penetrates the water column and decreases in intensity with depth. The absorbed heat is subsequently distributed by physical processes, including convection and wind-driven mixing. However, not all absorbed energy is retained, as part of it is lost to the atmosphere through long-wave radiation, evaporation, and convective heat transfer. These processes lead to thermal and chemical stratification, where surface layers often warm more rapidly than deeper layers, establishing a density gradient that influences vertical mixing patterns^6–10^.

Crater lakes are considered exceptional model systems. Typically formed within steep-walled volcanic depressions, their unique morphometry shields the water surface from wind, while their frequent hydrological isolation results in water balances that depend almost exclusively on direct exchanges with the atmosphere^11–15^. This combination of factors promotes strong and persistent density stratification, which is critical in regulating internal biogeochemical processes such as nutrient cycling and oxygen distribution^2,16–22^. Consequently, their isolation and energetically bounded nature make these lakes highly sensitive to external perturbations. They function as high-resolution sensors of atmospheric and hydrological processes, rendering them valuable sentinels of environmental change, particularly in fragile island ecosystems^23–25^.

**Fig. 1:**
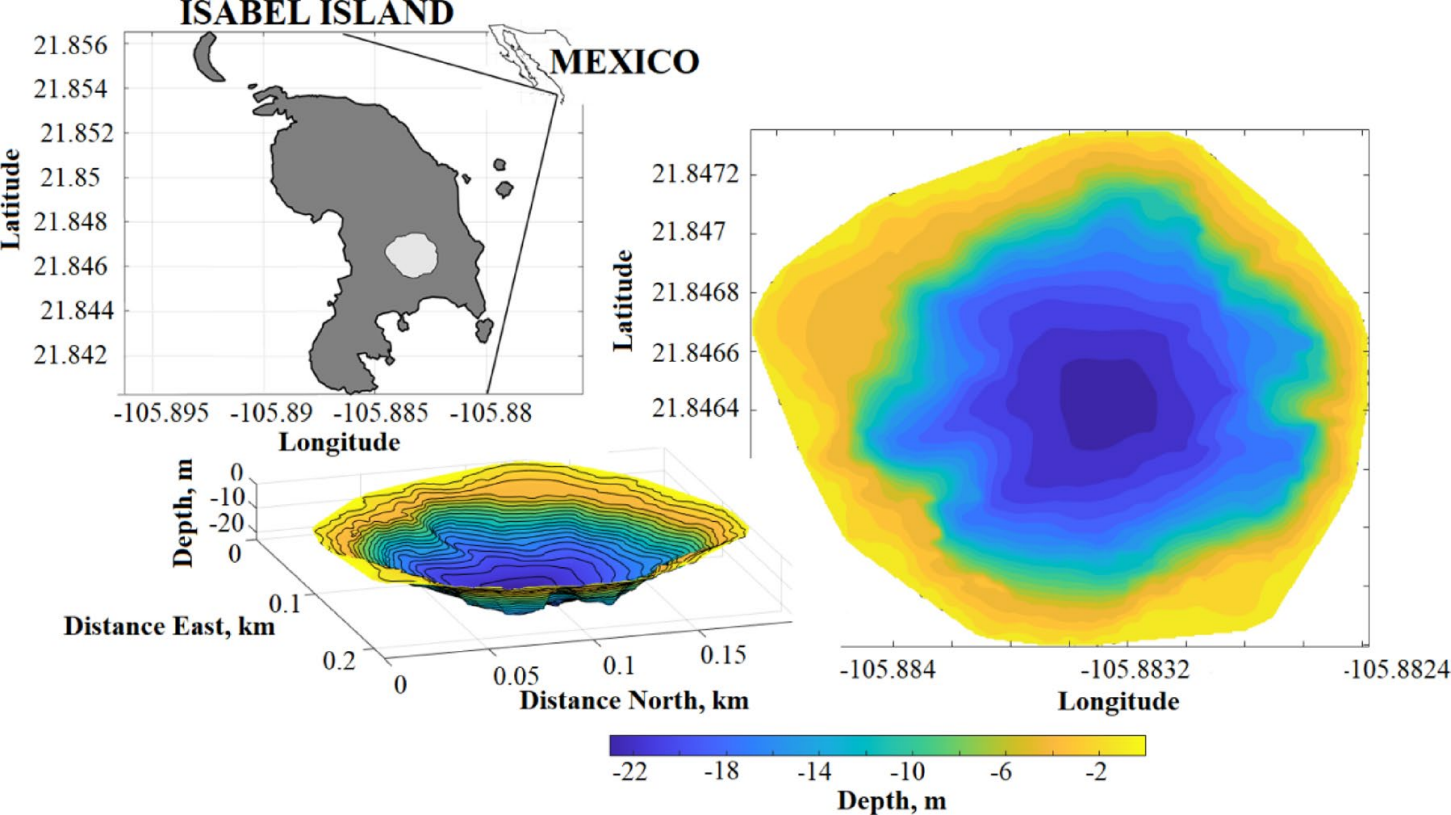
Geographical location of Isabel Island and its crater lake. Bathymetric map of the crater lake showing isobaths (in meters) in both horizontal and three-dimensional views.

The Isla Isabel crater lake, located in the Pacific Ocean off the coast of Nayarit, Mexico (Fig. 1), is an ideal system for investigating the interaction between meteorological, hydrological, and thermal processes in a closed and energetically bounded environment. Its circular morphology, geographic isolation, and persistent saline stratification (meromixis) make it a natural model for analyzing the surface energy balance. Although previous studies have attempted to estimate heat fluxes from the lake surface^26^, these were based on data collected at spatially and temporally separate stations, introduced significant uncertainty. This lack of synchronization limits the accuracy of thermal calculations, as it fails to integrate the key components regulating the lake’s thermal equilibrium in a simultaneous manner.

**Fig. 2:**
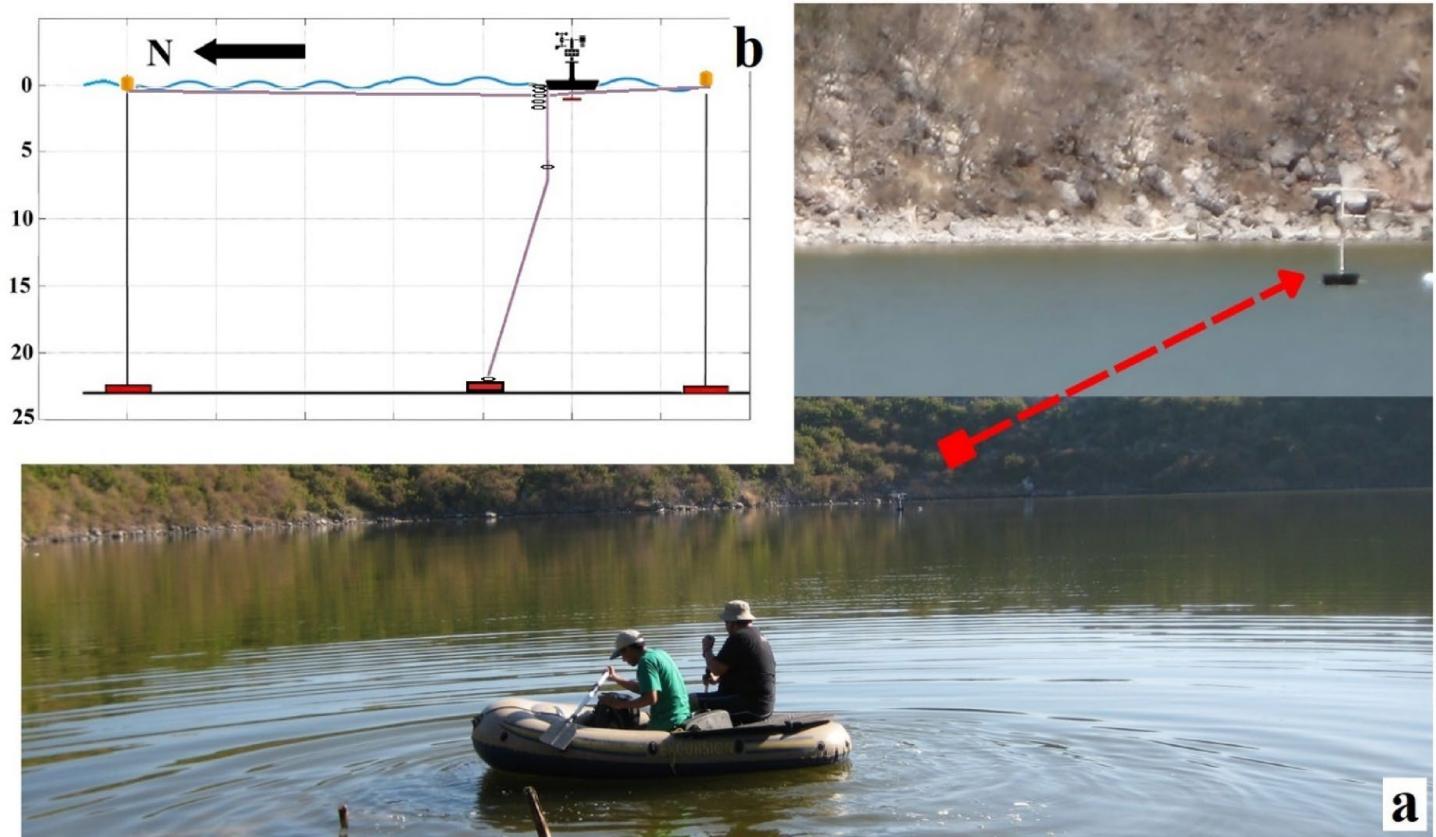
Monitoring system deployed in Isla Isabel crater lake. (**a**) Field image of the buoy installed at the center of the lake. (**b**) Schematic of the multiparameter buoy showing the general configuration of the anchoring system, sensors, and weather station.

The lake’s water is hypersaline^27^, with an approximate salinity of 80‰. The origin of this hypersalinity has been a subject of discussion. While some studies have suggested a possible connection to the sea through porous deposits^28^, other intensive field studies found no evidence of a tidal response, concluding that ongoing seawater intrusion is not a significant factor in the lake’s current water balance^29,30^. The consensus is that the lake’s high salt concentration is primarily the result of long-term evaporative processes acting on a hydrologically isolated water body^29,31^. The water also displays a very turbid olive-green color, with visibility measured by Secchi disc at only 50 cm (Fig. 2a), a condition likely caused by high concentrations of dissolved organic matter (DOM) and biogenic particles rather than inorganic sediments^26,27^.

The crater lake of Isla Isabel exhibits a strong and persistent chemical stratification, which defines two distinct layers with contrasting properties: a surface layer with low salinity and reduced dissolved oxygen, and a deep, hypersaline layer under permanently anoxic conditions. This stratification is also evident in the vertical pH profile, which ranges from alkaline values at the surface to neutral or slightly acidic conditions in the deeper layers^27,29^. Based on its characteristics, the lake is classified as a Type IV meromictic system^32^. This is supported by its morphometry, as the high crater walls (19–25 m) provide significant shelter from wind (Wedderburn number, W_e_ ≫ 1)^33^, and by the ectogenic origin of its salinity gradient.

Previous hydrographic data indicated that this stable water column allows for the development of an unusual thermal structure, including a sharp thermocline and a pronounced subsurface temperature maximum of approximately 35.7 °C at 1.69 m depth^26,34^. This subsurface warming is the defining characteristic of a heliothermal regime. While the principles of heliothermy are well-established, sustained heliothermal systems are remarkably rare, with “only about 30 hypersaline lakes worldwide that is meromictic and heliothermal"^35,36^. This positions Isla Isabel Lake as a key natural laboratory for investigating heat transfer in this uncommon class of aquatic environments.

The objective of this study is to quantify surface heat fluxes in the Isla Isabel crater lake through an integrated approach that combines in situ observations with numerical modeling, thereby providing a high-resolution, up-to-date assessment of the lake’s energy balance.

## Materials and methods

### Study area

The island Isabel is formed by a complex of coalescent volcanoes aligned with the Tepic-Zacoalco fault on the mainland. It is part of the province of alkaline-basaltic volcanic rocks that erupted during the Quaternary period^28^, and it is one of only two Mexican islands, together with Guadalupe, where the ascent of alkaline basalts to the surface has been documented. The total volume of the island is estimated at approximately 4.2 km³, of which only about 0.03 km³ emerges above sea level. The dominant lithology exposed is a cemented volcanic breccia of analcime-basalts, resulting from the rapid lithification of deposits generated by hydrovolcanic eruptions and lava flows.

A small crater lake is located in the southern portion of Isla Isabel, approximately 30 km off the Pacific coast of Nayarit, at coordinates 21°52′N and 105°54′W (Fig. 1). This geographical feature, documented in recent studies^26^, has a circumference of 816 m. The crater walls rise between 19 and 25 m above the water surface, which lies about 7 m above sea level. The bathymetry of the lake is characterized by a pronounced slope, with a maximum recorded depth of 23 m^26,27,31^.

According to the Köppen climate classification system, as modified by^37^, the Central Pacific region, where the island is located is characterized by a sub-humid tropical climate, primarily influenced by monsoon rains and tropical cyclones. Air temperatures remain warm to very warm throughout the year, with an annual average above 24.5 °C. The lowest mean temperature of 21.6 °C is recorded in February, which falls within the peak of the dry season. Conversely, the highest temperatures, reaching up to 30 °C, occur in October, during the transition from the rainy to the dry season, a period characterized by decreased cloud cover and high ocean heat content^26^.

The coastal region of Nayarit, where the island is located, experiences a tropical sub-humid climate influenced by the seasonal conditions of the Central Pacific. Relative humidity varies throughout the year, maximum values of up to 92.2% are recorded in September, coinciding with the peak of the rainy season. In contrast, minimum values of around 73.8% occur in January, which falls in the middle of the dry season. November is typically the driest month in terms of relative humidity, marking the transition between these two seasons.

On Isla Isabel and the surrounding coast, wind patterns show significant seasonal variation, which influences various physical processes such as wave formation, surface currents, sea breezes, and mountain flows^26^. During the dry season, prevailing winds come from the north-northwest, reaching speeds of up to 10 m/s. In the rainy season, wind directions become more variable, shifting between the northwest and northeast, with maximum speeds typically around 8.1 m/s in the afternoons. As the climate transitions back toward the dry season (October through December), the wind direction gradually shifts to the west and southwest, with intensities increasing later in the period to as high as 9.5 m/s. These atmospheric characteristics are key factors influencing the lake’s physical dynamics and thermal stability, as they regulate energy fluxes and heat exchange with the atmosphere.

### Isabel Island experiment

A multiparameter buoy was designed and constructed to enable the integrated recording of hydrometeorological variables. The buoy was built using a rectangular 85-liter container (1.20 × 0.40 × 0.50 m) filled with cold polyurethane foam, ensuring high buoyancy and structural stability. This platform served as the base for mounting the sampling equipment.

The installed instrumentation enabled the simultaneous recording of atmospheric and underwater variables. To measure temperature along the water column, four HOBO Temp Pro v2 Data Logger U22-001 thermistors were deployed at depths of 0.5, 1.5, 2.0, and 3.0 m, providing sufficient vertical resolution for thermal profiling of the lake. Additionally, HOBO U20 Water Level Data Loggers were placed at various depths (0.0 m, 7.0 m, and at the lake bottom) to monitor pressure and temperature concurrently. A CTD SBE16-PLUS sensor was positioned at a fixed depth of 1.0 m. This instrument continuously measured conductivity to monitor surface salinity changes and measured depth (via its pressure transducer) at an hourly resolution, allowing for the monitoring of surface salinity changes and precise tracking of variations in the lake’s water level. Water transparency was assessed using a 30 cm diameter Secchi disk, designed with alternating black and white quadrants to enhance visual contrast.

For atmospheric monitoring, the buoy was equipped with a HOBO RG3-M rain gauge capable of recording both air temperature and precipitation, as well as a HOBO S-WCA-M003 anemometer for high-accuracy measurements of wind speed and direction. It is important to note that while pyranometers for measuring shortwave solar radiation were also installed as part of the setup, the sensors were damaged during deployment and failed to record a usable dataset. Relative humidity was measured using a HOBO U23 Pro v2 sensor, while a DMA-4500 density meter was used to determine water density, from which a single vertical profile of the water column was obtained.

The weather station was mounted on the buoy using plastic and PVC components. A central mast extended 1.5 m above the lake surface and 1 m below, serving both to support the atmospheric sensors and to anchor the structure. To maintain the buoy’s stability and fixed position, a dead weight of approximately 40 kg was placed at the lake bottom and connected to the submerged sensor line. Additional protective measures were implemented to prevent interference from large birds, which are commonly found at the study site.

The lake under study is an endorheic and hypersaline system with adiabatic boundaries, meaning there is no internal heat generation and that energy and mass exchanges occur exclusively with the external environment. This condition, combined with its geographic setting, makes the lake function as a natural pluviometer, where water accumulation and intense evaporation lead to strong stratification and elevated salinity levels over time^17^. The density profile was determined through in situ sampling and densimeter analysis, complementing the physicochemical characterization of the system.

The buoy was deployed at the center of the lake and operated continuously for nearly one year, recording hourly data for all instrumented variables. The robustness of its design ensured the stability and uninterrupted operation of the station even during adverse weather conditions, resulting in near-complete data coverage and high-quality measurements for subsequent scientific analysis.

### Field observations

The time series for wind speed, atmospheric pressure, relative humidity, precipitation, air temperature, and water column temperature were obtained using instruments programmed for hourly resolution, covering the period from March 23, 2011, to February 14, 2012. The 11-month duration of the time series was determined by the equipment retrieval date authorized by the federal agency (PROFEPA) that manages access to the protected national park. The temperature sensor array, anchored to the buoy station (Fig. 2b), was positioned at the following depths below the water surface: 0.0, 0.5, 1.0, 1.5, 2.0, 3.0, 4.0, 7.0 m, and at the lake bottom. Pressure sensors were installed along the same vertical line at depths of 0.0, 1.0, 7.0 m, and at the bottom.

The instruments used to measure relative humidity, wind speed, and precipitation were installed on a mast approximately 1.5 m tall. On the other hand, the sensors for precipitation, air pressure, and air temperature were positioned about 1.30 m above the water surface. The sensors for humidity and wind speed eventually stopped functioning due to battery depletion, resulting in a data gap. This gap was filled by correlating the truncated time series with data from the North American Regional Reanalysis (NARR), available through the NOAA website (http://www.emc.ncep.noaa.gov/#narr_datasets). Cloud cover data, also obtained from NOAA, were available at 24-hour intervals. It is important to note that while pyranometers for measuring shortwave solar radiation were also installed as part of the setup, the sensors were damaged during deployment and failed to record a usable dataset.

To prepare the data for heat balance analysis at the lake-atmosphere interface, a low-pass Godin filter^38^ was applied to remove the diurnal signal, as the model operates with a daily resolution time series. A single, high-resolution vertical density profile was measured during the field campaign to characterize the baseline haline stratification of the lake. Water samples were collected at 1-meter intervals, and each sample was measured three times using a densimeter, with the third reading recorded as the final value. All collected data were plotted and found to be consistent in magnitude and seasonal trends with those reported by ^26^, confirming the reliability of the dataset for subsequent analysis.

### Heat budget model description

The one-dimensional model was employed not as a predictive tool, but as a diagnostic tool to verify whether the measured surface heat fluxes could account for the fundamental thermal structure observed in the lake, particularly the formation of the heliothermal layer.

The heat conservation model applied in this study is one-dimensional, based on the assumption that temperature is horizontally homogeneous in the upper layer of the water body^39^. Accordingly, the heat conservation equation used is that described by Simpson and Dickey^40^:

1 $$\:\frac{\partial\:T}{\partial\:t}+\frac{\partial\:}{\partial\:z}\left(\stackrel{-}{W{\prime\:}T{\prime\:}}\right)=\frac{1}{\rho\:{C}_{p}}\frac{\partial\:I}{\partial\:z}$$

where is temperature, $\:t$ is time, the vertical turbulent kinematic heat flux (the time-averaged product of the turbulent fluctuations of vertical velocity, $\:W{\prime\:}$, and temperature ), $\:\rho\:$ is the in-situ density, is the specific heat of seawater, and according to^41^, $\:I$ is the penetrative fraction of total solar radiation, represented by the following expression:

2 $$\:I={I}_{0}{e}^{\gamma\:z}$$

So $\:{I}_{0}$ the radiation reaches the water surface and decays exponentially as a function of depth $\:\left(z\right)$, $\:\gamma\:$ the extinction coefficient that varies between 3 and 10 m ^42^. However, as it is empirically related to the depth to which the Secchi disk is visible, it can be modified due to the water turbidity.

Substituting Eq. (2) in Eq. (1), the following expression is obtained:

3 $$\:\frac{\partial\:T}{\partial\:t}+\frac{\partial\:}{\partial\:z}\left(\stackrel{-}{W{\prime\:}T{\prime\:}}\right)=\frac{1}{\rho\:{C}_{p}}\frac{\partial\:{I}_{0}{e}^{\gamma\:z}}{\partial\:z}$$

Since the temperature and solar radiation penetration functions are considered to be continuous in the vertical, Eq. (3) can be integrated from $\:z=-h$ to $\:z=0$

4 $$\:\frac{\partial\:T}{\partial\:t}h+{\left.\left(\stackrel{-}{W{\prime\:}T{\prime\:}}\right)\right|}_{z=0}-{\left.\left(\stackrel{-}{{W}^{{\prime\:}}{T}^{{\prime\:}}}\right)\right|}_{z=h}=\frac{{I}_{0}\left(1-{e}^{-\gamma\:h}\right)}{\rho\:{C}_{p}}$$

Turbulent surface fluxes, according to^42^, can be described in terms of heat fluxes across the surface:

5 $$\:\left(\stackrel{-}{{W}^{{\prime\:}}{T}^{{\prime\:}}}\right)=\frac{{Q}_{f}}{\rho\:{C}_{p}}$$

where $\:{Q}_{f}$ is expressed as:

6 $$\:{Q}_{f}={I}_{0}-{Q}_{e}-{Q}_{b}-{Q}_{h}$$

$\:{Q}_{e}$ is the radiation energy emitted by the surface as if it behaved as a black body, $\:{Q}_{b}$ the latent heat flux energy, and $\:{Q}_{h}$ the sensible heat flux energy. These fluxes are represented and calculated based on the atmospheric conditions of the study site.

As a necessary simplification for this bulk heat budget model, it is assumed that the mixing layer depth, $\:h$, remains constant. This assumption implies that the turbulent flow conditions at the bottom of the layer are zero, which allows Eq. (4) to be reduced to:

7 $$\:\frac{\partial\:T}{\partial\:t}=\frac{{I}_{0}\left(1-{e}^{-\gamma\:h}\right)}{\rho\:{C}_{p}h}-\left(\frac{{I}_{0}-{Q}_{e}-{Q}_{b}-{Q}_{h}}{\rho\:{C}_{p}h}\right)$$

If it is considered that the radiation reaching the surface of the water is distributed throughout the water column, i.e., $\:-\gamma\:h=0$ then Eq. (7) would be:

8 $$\:\frac{\partial\:T}{\partial\:t}h-\left(\frac{{I}_{0}-{Q}_{e}-{Q}_{b}-{Q}_{h}}{\rho\:{C}_{p}}\right)=0$$

The recorded atmospheric conditions, were used as model forcing, which estimates heat fluxes across the surface and predicts the temporal evolution of temperature. As direct measurements of solar radiation were unavailable due to the previously noted sensor failure, incident shortwave radiation was estimated using established bulk formulae based on measured cloud cover, other atmospheric variables, and reanalysis data. In addition, the initial temperature in the update of each step of the model run was forced to be the observed surface temperature; this was done to simulate the decrease in the surface temperature due to a large amount of water brought in during the rainy season.

All heat fluxes were calculated using the heat balance equations for an unfrozen lake, originally proposed by Lavin and Organista^42^ and later applied by Palacios-Hernández, et al.^26^. These calculations require surface water temperature, air temperature, relative humidity, atmospheric pressure, and wind speed. Unlike the flux estimates in Palacios-Hernández, et al.^26^, this study employs simultaneous, in situ measurements, which enhances the reliability of the results. Model outputs were visually compared with temperature profiles obtained from thermistor observations.

### Water column stability

The degree of mixing and stratification in the water column was quantified using the Schmidt Stability Index (SSI), which represents the amount of energy required to achieve complete mixing. This index offers valuable insight into the temporal variability of the density structure in stratified systems^22,43–45^. The calculation of daily SSI requires a time series of vertical density profiles. These profiles were computed using the official thermodynamic equation of state for seawater, TEOS-10, which is suitable for high-salinity waters (105 mS cm⁻¹ ≈ 78‰; Kienel, et al.^31^), The calculations were implemented with the Gibbs SeaWater (GSW) toolbox^46^.

The TEOS-10 calculation requires inputs of Absolute Salinity, Conservative Temperature, and pressure. The continuous time series of temperature profiles from our thermistor chain provided the necessary temperature and pressure data. However, as continuous conductivity was only measured at 1 m depth by the CTD SBE16-PLUS sensor, a time series of full-depth salinity profiles was estimated as follows: First, a single, high-resolution vertical profile of the lake’s salinity structure, measured in situ, was used to characterize the stable shape of the deep-water halocline. Second, the continuous data from the 1 m sensor was used to dynamically adjust the salinity of the upper mixolimnion to reflect seasonal changes, such as dilution from rainwater.

This approach assumes that the shape of the halocline in the deep, isolated monimolimnion remains constant, which is a reasonable assumption for a permanently stratified meromictic system. The resulting estimated daily density profiles were then used to compute the daily SSI values.

The Schmidt Stability Index (SSI) is calculated using the formulation originally described by Schmidt^44^ as follows:

9 $$\:SSI=\frac{g}{A}{\int\:}_{0}^{{z}_{D}}\left(z-{z}_{v}\right){\rho\:}_{z}{A}_{z}dz$$

where $\:g$ is the acceleration due to gravity, $\:{\rho\:}_{z}$ is the water density at depth $\:z$, $\:{A}_{z}$ is the cross-sectional area of the lake at depth $\:z$, $\:{z}_{D}$ is the maximum depth of the lake, and $\:{z}_{v}$ is the depth of the center of volume of the lake, calculated as:

10 $$\:{z}_{v}=\frac{{\int\:}_{0}^{{z}_{D}}z{A}_{z}dz}{{\int\:}_{0}^{{z}_{D}}{A}_{z}dz}$$

The temporal evolution of thermal stability was examined on both daily and monthly scales. Model accuracy was assessed using the systematic error (bias or BIAS) and the root mean square error (RMSE)^47,48^.

## Results

### Meteorological conditions

**Fig. 3:**
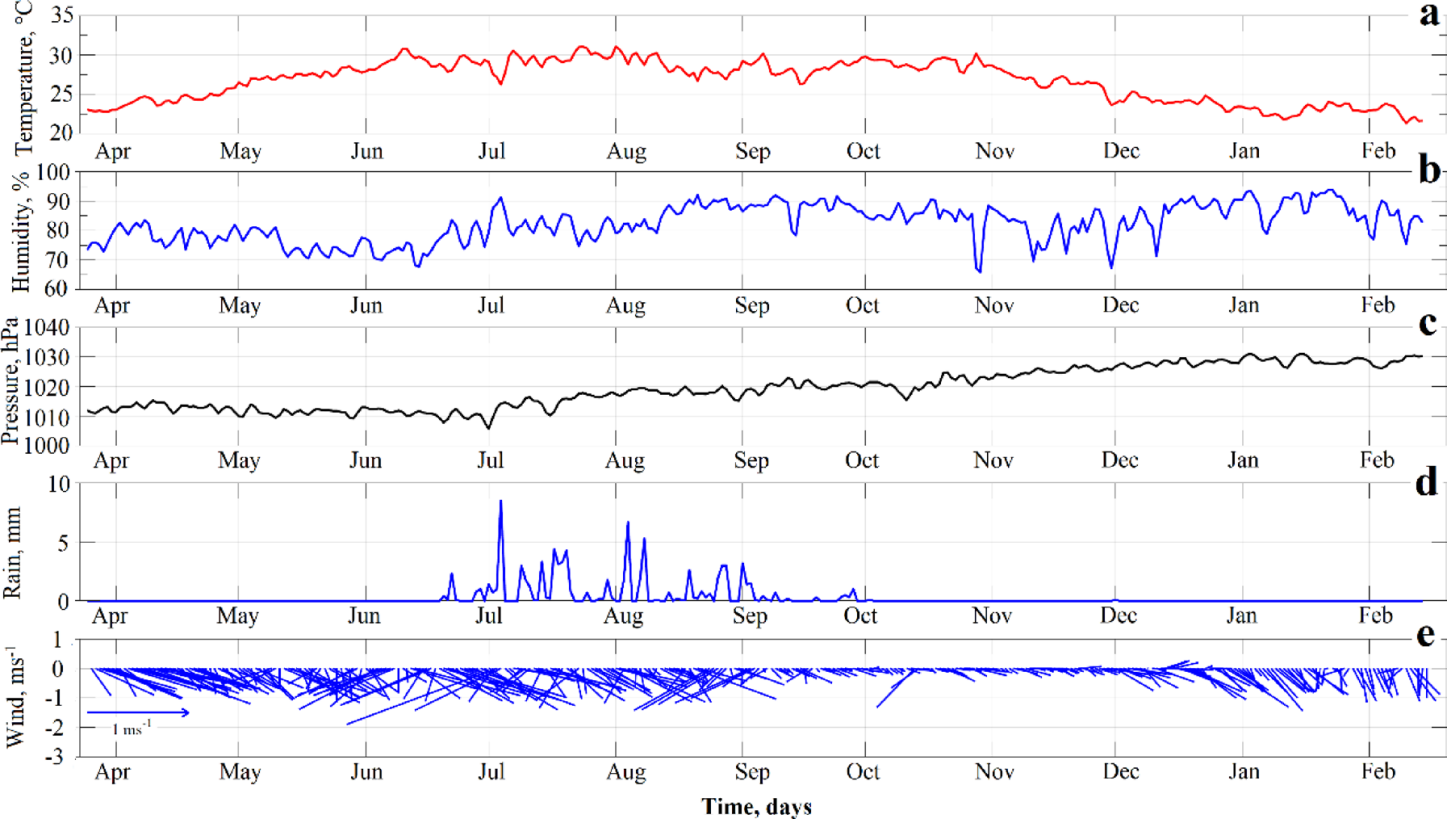
Meteorological observations from Isla Isabel: (**a**) air temperature, (**b**) relative humidity, (**c**) atmospheric pressure, (**d**) precipitation, and (**e**) wind, recorded from 27 March 2011 to 16 February 2012.

The time series of the main meteorological variables recorded in situ illustrate the region’s seasonal dynamics (Fig. 3). The air temperature shows a well-defined seasonal pattern, rising from the dry season to a peak of approximately 35 °C during the rainy season, before declining toward the following dry period (Fig. 3a). The surface temperature of the lake follows a similar trend, with the difference between air and water temperatures reaching up to 5 °C early in the dry season and decreasing to an average of around 2 °C as the rainy season progresses. Relative humidity remained consistently high throughout the study period, with values exceeding 70% (Fig. 3b). Concurrently, a sustained decrease in atmospheric pressure was observed from April (~ 1035 hPa) to September (~ 1005 hPa), followed by a gradual increase (Fig. 3c). The rainy season was marked by intense and frequent precipitation events between July and August, where daily peaks reached nearly 9 mm (Fig. 3d).

Winds measured at the center of the lake were predominantly weak, with maximum speeds reaching up to 2 m/s, showing greater variability and intensity during the rainy season (Fig. 3e). A comparison with data from the Isla Isabel lighthouse station revealed significant differences. Air temperature at the center of the lake was consistently between 1.5 and 4.2 °C higher, relative humidity was approximately 5% higher, and wind speeds were between 1.5 and 3 m/s lower than those recorded at the lighthouse.

### Seasonal thermal structure

**Fig. 4:**
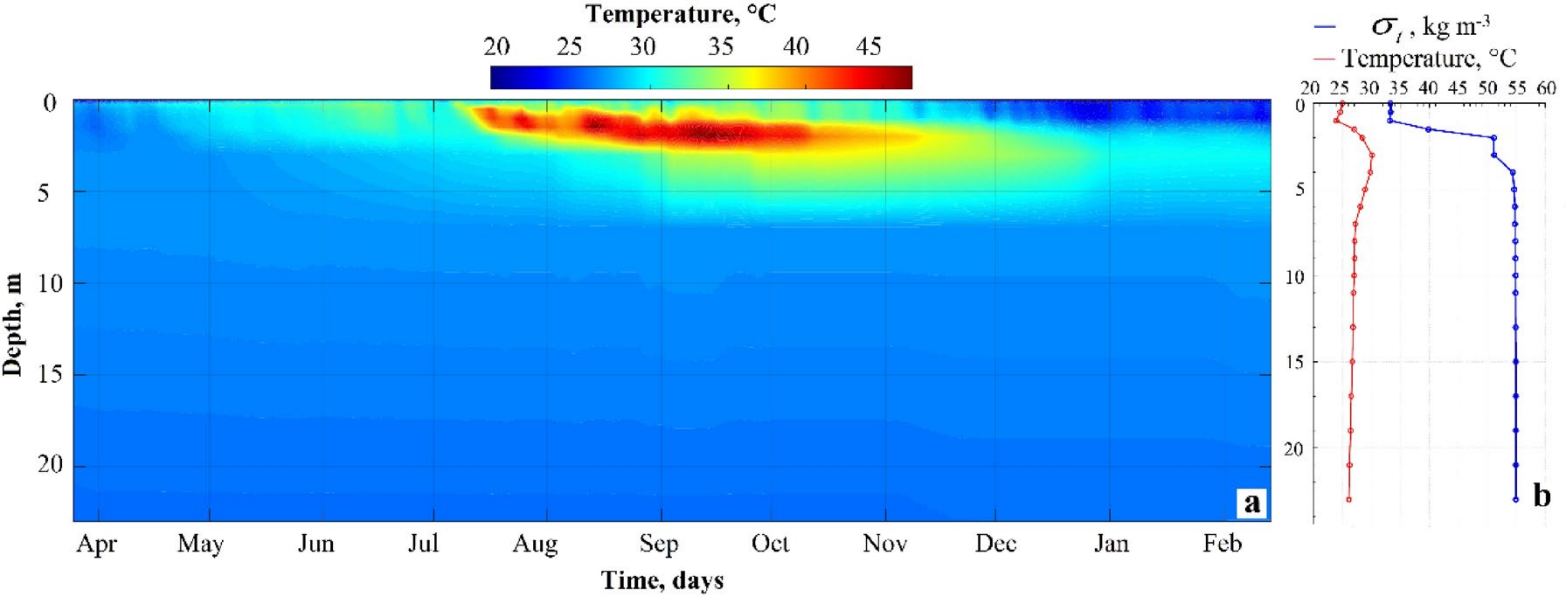
Thermal and density structure of the Isla Isabel crater lake. (**a**) Seasonal evolution of the vertical temperature profile (°C) from March 2011 to February 2012, highlighting the formation of the subsurface heliothermal layer. (**b**) Vertical profiles of temperature (°C; red curve) and the potential density (*σ*_*t*_; blue curve) from on 14 February 2012.

The lake’s thermal structure exhibited a distinct seasonal pattern (Fig. 4a). During the dry season (March to mid-May), the mixolimnion showed relatively uniform temperatures between 27 °C and 31 °C. The onset of seasonal thermal stratification began in late May. During the rainy season (June to September), the lake underwent pronounced surface heating, with temperatures in the mixolimnion exceeding 40 °C between 0.5 and 5 m depth and reaching peak values close to 47 °C (Fig. 4a). Toward the end of the rainy season and into the dry season (October to February), the mixolimnion underwent progressive surface cooling, and thermal stratification become less pronounced. Throughout the entire year, the deep layers (monimolimnion, ≥ 10 m) remained thermally stable, with temperatures consistently ranging between 26 and 27 °C.

The vertical profiles of temperature and potential density (σₜ) measured on February 14th 2012, further details the stratified structure (Fig. 4b). The uppermost layer (0–1 m) exhibited a thermal inversion, with temperatures increasing from about 25 °C at the surface to nearly 30 °C at 5 m depth. Simultaneously, potential density (σₜ) rises sharply from 34 to 35 kg m⁻³ to approximately 51 kg m⁻³. The intermediate zone (4–6 m) was characterized by a moderate decrease in temperature (from 30 °C to 28 °C) and a pronounced increase in density (σₜ from 51 to 56 kg m⁻³), forming the lake’s dominant pycnocline. The monimolimnion, from 7 m to the bottom (~ 23 m), exhibited a nearly constant temperature (25.5–26 °C) and stable density (σₜ ~56 kg m⁻³).

### Surface heat fluxes and energy balance

**Fig. 5:**
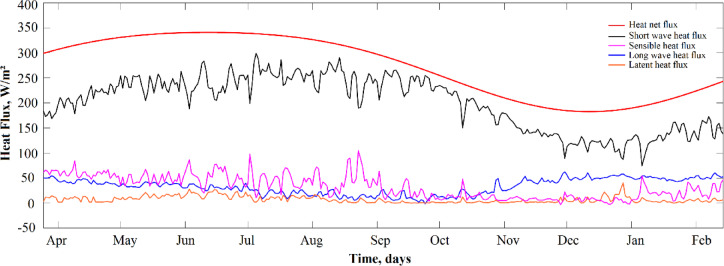
Daily heat flux components (in W m⁻²) in Isla Isabel crater lake from March 2011 to February 2012. Short wave radiation flux (red), long wave radiation flux (blue), latent heat flux (orange), sensible heat flux (magenta), and heat storage in the lake (black).

The daily heat fluxes at the surface lake were estimated from the period from 25 March 2011 to 14 February 2012 (Fig. 5). Shortwave solar radiation, the primary source of heating, displayed a distinct seasonal pattern, with maximum values approaching 345 W/m² during the rainy season and minimums falling to approximately 190 W/m² during the dry season. Longwave radiation showed lower seasonal variability, with daily fluxes ranging between 25 and 55 W/m². The latent heat flux ranged from 10 to 30 W/m², with peak values in the rainy season. The sensible heat flux exhibited strong seasonal variability, with episodic peaks reaching up to 100 W/m².

The total energy balance reaches its highest values between March and early October, with a rainy season maximum of approximately 290 W/m². From October onward, the energy budget declined steadily, reaching its lowest values of 100 and 130 W/m² during the dry season.

### Modeled thermal evolution and stability

**Fig. 6:**
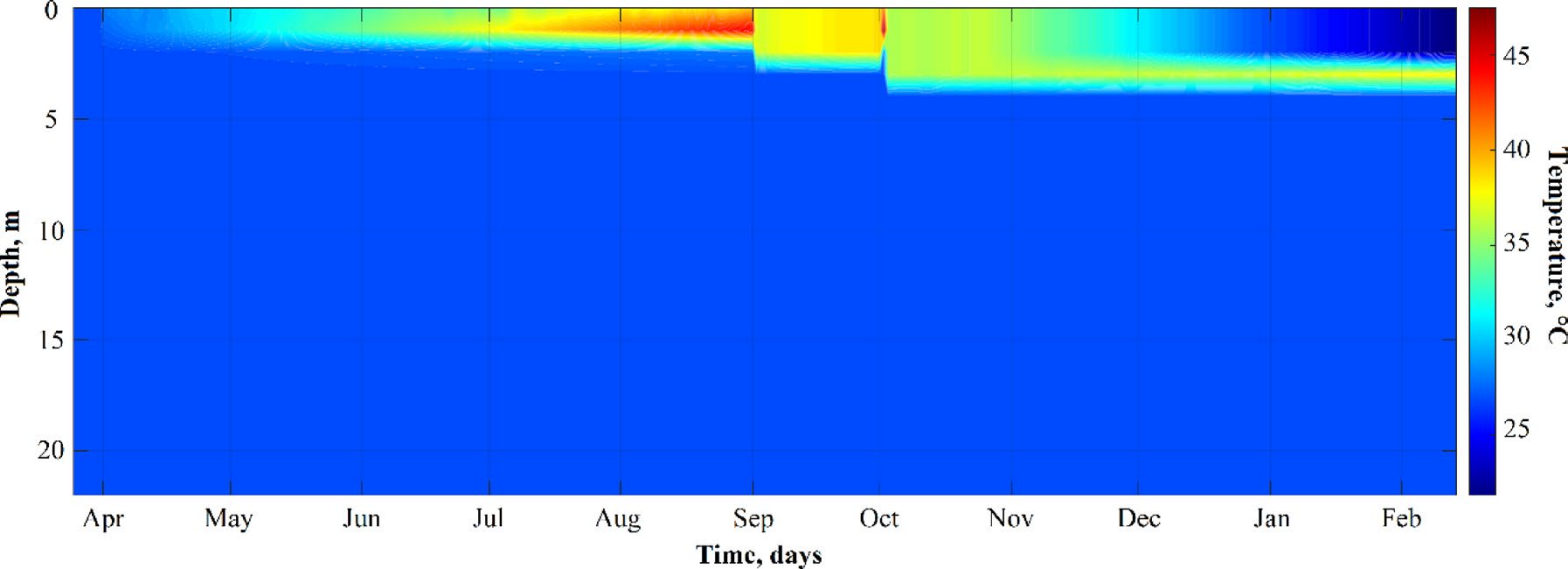
Simulated vertical temperature profile in the water column of Crater Lake, Isabel Island, for the period from 27 March 2011 to 16 February 2012.

The temporal evolution of temperature with depth, as simulated by the one-dimensional heat balance model, is presented in Fig. 6. The model reproduced the overall thermal structure, including the formation of the heliothermal layer between 2 and 5 m depth, and simulated the seasonal progression of thermal stratification and its gradual weakening. An abrupt surface cooling event was simulated in early October by applying a daily update of observed surface temperatures.

**Fig. 7:**
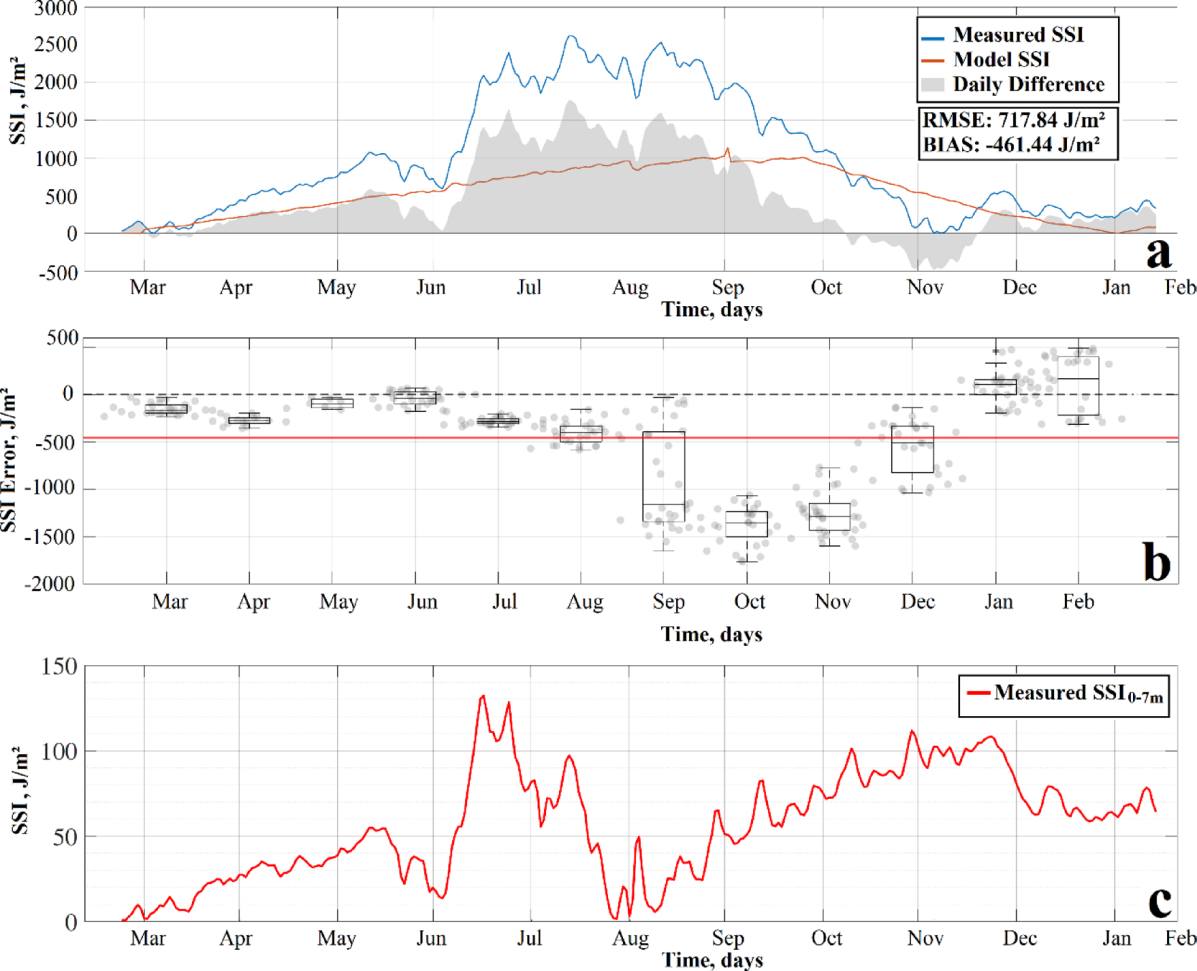
Comparison between observed and modelled Schmidt Stability Index (SSI) in Isla Isabel crater lake from 25 March 2011 to 14 February 2012. (**a**) Time series of accumulated SSI: observed (blue), modelled (red), and daily difference (grey); the shaded area represents the monthly distribution of daily SSI error. (**b**) Monthly root mean square error (RMSE) between modelled and observed SSI. (**c**) Time series of observed SSI calculated for the top 7 m of the water column (S₀₋₇ₘ).

The Schmidt Stability Index (SSI), calculated for the entire water column form observed and modeled temperature profiles, showed a high degree of temporal agreement (Fig. 7a). During the dry season, the SSI displayed a moderately increasing trend, reaching values close to 1000 J/m². As thermal stratification intensified during the rainy season, the whole-column SSI reached its peak values of approximately 2700 J/m²). In October, an abrupt drop in the whole-column SSI was observed. The global root mean square error (RMSE) between the measured and modeled SSI was 717.84 J/m², with a negative mean bias of −461.44 J/m². The monthly distribution of daily SSI errors is presented in Fig. 7b.

The SSI calculated for the top 7 m (S₀₋₇ₘ), revealed a more volatile seasonal pattern (Fig. 7c), increasing from a minimum of 0.45 J/m² in late March to a seasonal peak of 132.1 J/m² on July 17, followed by high variability during the rainy season.

### Haline control of seasonal thermal stratification

**Fig. 8:**
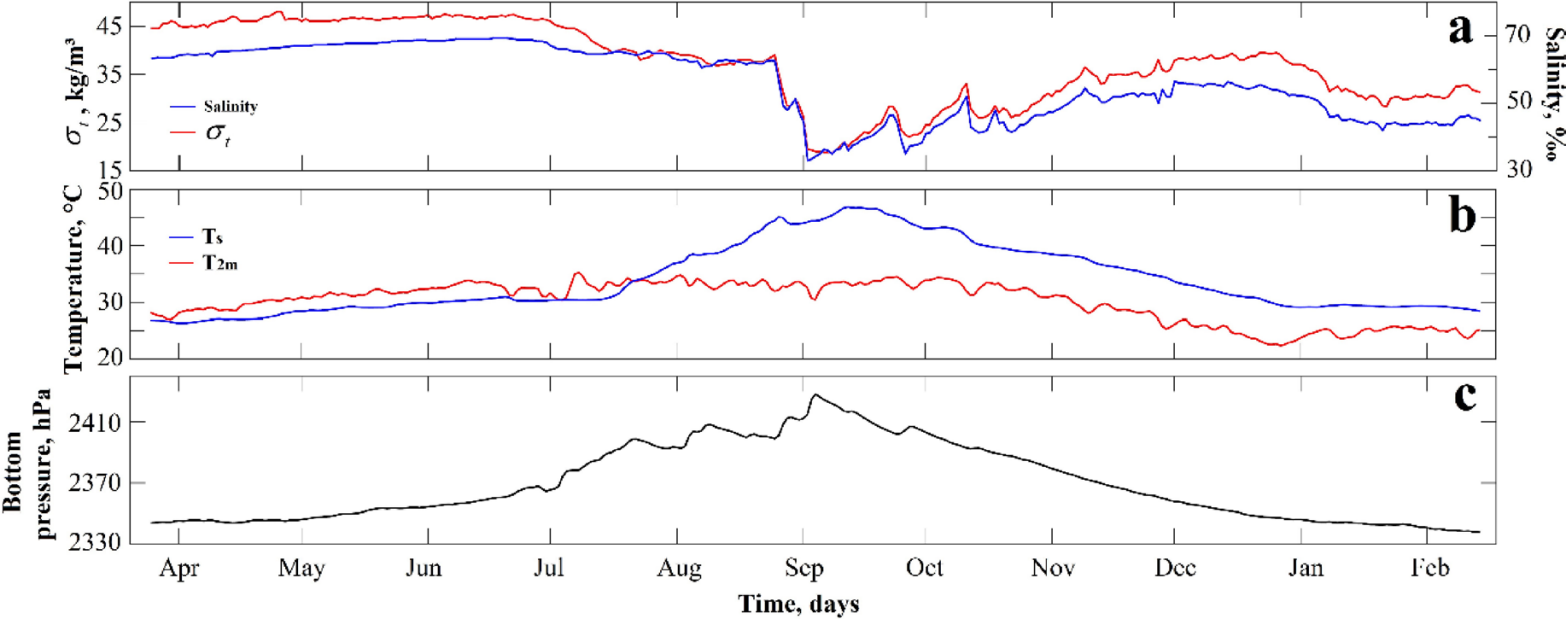
Temporal evolution of physical variables in Isla Isabel crater lake from 25 March 2011 to 14 February 2012. (**a**) Salinity (blue) and potential density σₜ (red) at 1 m depth. (**b**) Surface temperature (red) and temperature at 2 m depth (blue). (**c**) Bottom pressure, calculated as the difference between absolute and atmospheric pressure.

The relationship between salinity and potential density (σₜ) at 1 m depth was exceptionally strong (Fig. 8a), with a Pearson correlation coefficient of 0.9629 (Table 1; *p* < 0.001). From April to late August, surface salinity values were stable, ranging between 60 and 65‰, accompanied by a decline in potential density. These values did not return to their pre-dilution levels during the remainder of the observation period. The time series of surface and subsurface temperatures reveals a well-defined annual thermal cycle (Fig. 8b). From March to mid-July, surface temperature was consistently 2 and 3 °C higher than the temperature at 2 m depth. Beginning in mid-July, this dynamic shifted, and the subsurface layer become warmer than the surface. The bottom pressure sensor, which acts as a high-precision lake-level logger, reveals a clear seasonal pattern in the lake’s total water mass (Fig. 8c).

**Table 1:** Pearson correlation coefficients (r) and associated p-values from the linear correlation analysis between surface physical variables (temperature at the surface and at 2 m depth, salinity and σₜ at 1 m) and the bottom pressure gradient.

	T_sup_	T_2m_	Salinity	σₜ	Bottom pressure
T_sup_	1	0.5241	0.2264	0.0115	0.7145
T_2m_	0	1	−0.5676	−0.7288	0.9039
Salinity	0	0	1	0.9629	−0.3231
σₜ	0	0	0	1	−0.5447
Bottom pressure	0	0	0	0	1

**Fig. 9:**
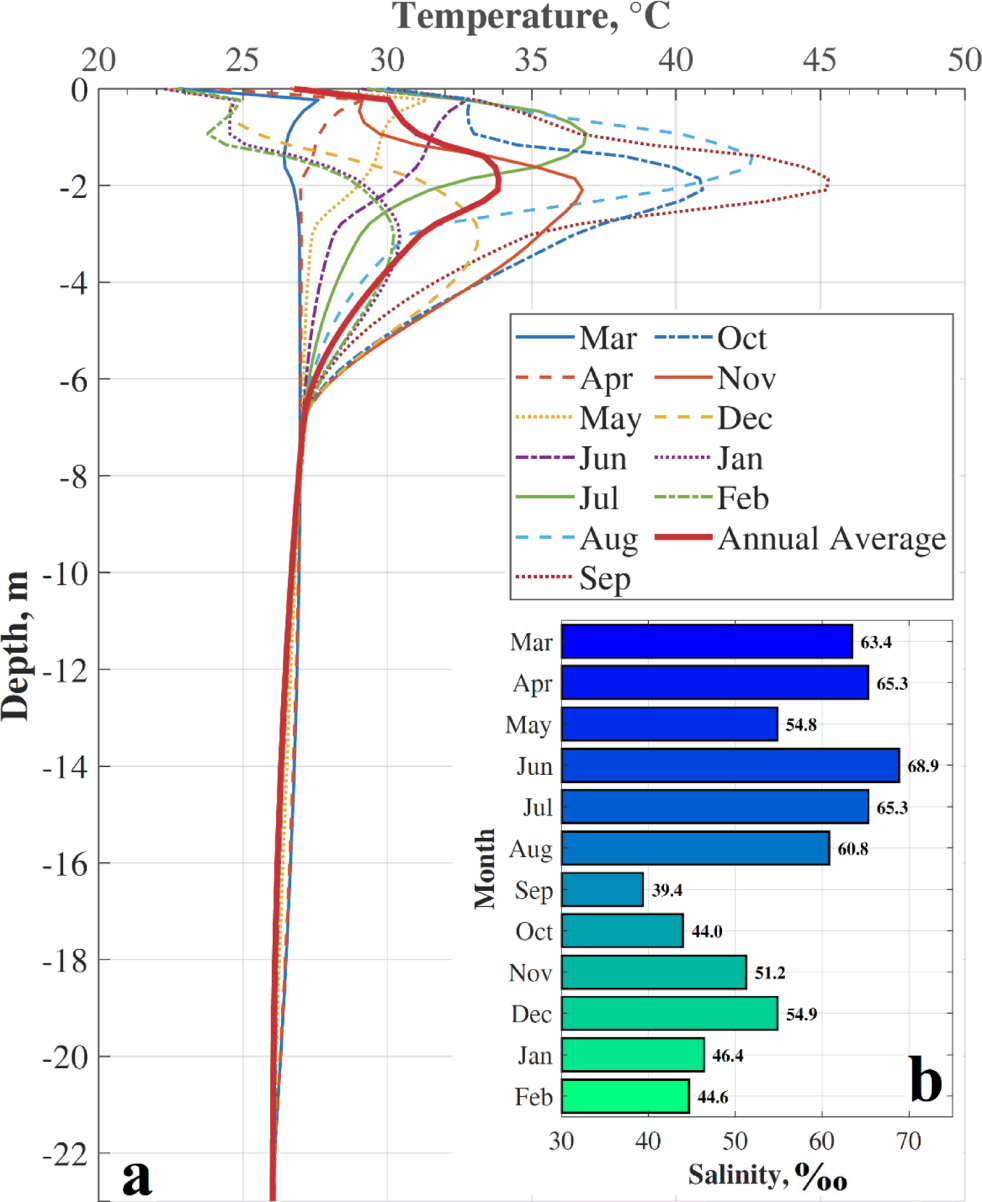
Seasonal thermohaline dynamics in the mixolimnion from March 2011 to February 2012. (**a**) Monthly-averaged temperature profiles. The solid red line represents the annual average. (**b**) Corresponding monthly averages of salinity measured at 1 m depth.

The monthly-averaged temperature profiles reveal a system that alternates between two distinct thermal states (Fig. 9a). During the extended dry season (approximately from November to May), the profiles show a nearly isothermal mixolimnion. In sharp contrast, with the onset of the rainy season, the profiles evolve to show a pronounced subsurface thermal maximum. This heliothermal layer intensifies to its peak in September, when the temperature at ~ 2 m depth is more than 15 °C warmer than the surface. The annual average profile (solid red line) identifies the mean position of this heliothermal feature at 1.86 m.

The primary driver for this thermal transition is shown in Fig. 9b. The well-mixed thermal state corresponds directly to a period of high and stable surface salinity (~ 65‰). The rapid development of intense thermal stratification coincides perfectly with the major surface dilution event, where salinity drops abruptly by nearly 30‰.

## Discussion

### The lake’s microclimate and its influence on surface energy dynamics

The results demonstrate a strong thermal coupling between the atmosphere and the lake surface. However, the specific observations from this study reveal both regional and local processes governing the system. The recorded barometric trend, for instance, aligns with the regional dynamics influenced by the Mexican monsoon and the hurricane season, as detailed by Amador, et al.^49^. At more local scale, wind direction showed greater variability and intensity during the rainy season, coinciding with seasonal peaks in precipitation, with suggests a coupling between hydrological processes and wind dynamics the crater^26,50^.

The clearest evidence of these localized conditions comes from the comparison between the meteorological records from this study and those reported by Palacios-Hernández, et al. ^26^ for the station at the Isla Isabel lighthouse. The significant differences, including consistently higher air temperatures, greater humidity, and lower wind speeds over the lake, confirm the formation of a distinct microclimate within the crater. This sheltered environment directly impacts the surface energy balance, where the dominance of shortwave radiation as the primary energy input is the key driver of the intense surface heating observed. This dynamic, where seasonal climatic forcing induces significant variability in thermal structure, is consistent with observations in other tropical and temperate lakes^51^, highlighting that even in a highly stable meromictic system, atmospheric forcing governs the thermal evolution.

### Drivers of seasonal stratification and morphological controls

A key finding from the thermal analysis is the remarkably short duration of strong thermal stratification within the mixolimnion. As shown in the results, this condition persists for only approximately five months (late May to early October), corresponding to the rainy season. For the majority of the year, the ~ 4 m deep mixolimnion remains in a well-mixed, nearly isothermal state. This seasonal pattern, characterized by a prolonged mixed period, contrasts sharply with many deep tropical lakes where thermal stratification is a quasi-permanent, year-round feature^9,12,15,52–55^. The primary reason for this difference likely lies in the morphometry of Isla Isabel Lake and the shallow depth of its mixolimnion. A thin upper layer possesses low thermal inertia, making it highly susceptible to nocturnal convective cooling and wind-driven mixing, particularly during the dry season when the stabilizing effect of freshwater input is absent. This constant erosive energy appears sufficient to overcome diurnal solar heating and maintain a mixed state for over half the year, a dynamic that highlights the critical interplay between basin morphology and atmospheric forcing in controlling the stability of tropical lakes.

### Evaluating a thermal model in a heliothermal, meromictic system

The one-dimensional model successfully captures the principal seasonal thermal dynamics of the system, emphasizing the dominant role of solar radiation, evaporative cooling, and surface heat exchange in governing the lake’s thermal structure. However, its limitations reveal the central role of salinity. The primary limitation is its inability to account for changes in salinity and their dominant effect on density. The omission of salinity-induced density gradients is the main reason the model struggles to accurately reproduce the extent of vertical mixing and, consequently, the SSI, particularly during and after the rainy season^56–58^. This is evident in the model’s systematic underestimation of the whole-column stability, reflected by the negative mean bias in the SSI.

While the omission of salinity reduces the precision of stability forecasts, the model provides a valuable first-order approximation of the vertical thermal dynamics. By qualitatively reproducing the formation, maintenance, and decay of the heliothermal layer, the model allows for a preliminary analysis of the thermal response to atmospheric forcing. It is fundamental to interpret these results within this context: the model serves to identify general trends and the basic thermal structure, and any conclusions regarding stability must be interpreted with caution. Although more complex models could provide greater accuracy, the current approach offers the advantage of simplicity, effectively explaining the formation of the heliothermal layer without losing this key feature in additional complexity.

### Evaluating a thermal model in a heliothermal, meromictic system

The seasonal dynamics presented in the results provide a clear, quantitative demonstration of how haline forcing at the surface directly controls the formation, intensification, and eventual decay of the heliothermal layer. This confirms a classic mechanism of ectogenic meromixis, where external freshwater input is the primary agent reinforcing stratification, characteristic of Type IV systems as defined by Walker and Likens³². The abrupt drop in salinity creates a strong, shallow pycnocline, or freshwater cap, at the surface. This low-density lid effectively suppresses vertical mixing, acting as a physical barrier that traps incoming solar radiation in the layers immediately below.

The mechanism of heat absorption within this trapped layer is also critical. The phenomenon observed, where the subsurface layer becomes significantly warmer than the surface, indicates that solar radiation is being absorbed efficiently at depth. This is not due to bottom heating, as seen in classic shallow solar ponds, but rather occurs within the water column itself. The low Secchi depth (0.5 m) strongly suggests the presence of an optically active layer, an inference directly supported by the findings of Kienel, et al. ^31^, who previously documented a distinct turbidity maximum from a thin layer of photosynthetic bacteria at the chemocline in this very lake. This optically active layer is therefore the primary engine responsible for absorbing solar radiation and driving the intense heliothermal heating.

Furthermore, the high-resolution pressure data provides an independent confirmation of the system’s response (Fig. 8c). The strong positive correlation between bottom pressure and the subsurface temperature (*r* = 0.9039) is consistent with volumetric thermal expansion of the water column as this immense heat is stored. Conversely, the negative correlation with surface salinity (*r* = − 0.3231) illustrates the dual role of precipitation: it increases the total water mass (raising pressure) while simultaneously diluting the surface, which is the very action that enables the heat to be trapped.

The fundamental mechanism is analogous to that of other well-known heliothermal systems, such as the Pretoria Salt Pan in South Africa^34^, Lake Hayward in Australia^36^, and Pulicat Lagoon in India^59^. However, the pronounced seasonality and the extended period of isothermal conditions in the mixolimnion highlight a distinct annual cycle for this tropical crater lake.

Ultimately, this study provides a high-resolution demonstration of a sustained heliothermal regime in a hypersaline, meromictic lake, a contribution that addresses a significant gap in limnological knowledge. Meromictic lakes themselves are uncommon, and those that also exhibit heliothermal behavior are exceptionally rare, numbering “less than 30 worldwide"^35,36^. Therefore, this detailed characterization of the Isla Isabel crater lake not only adds to the limited body of knowledge on this rare class of lake but also establishes it as a model system for investigating heat transfer processes and physical stability under extreme tropical conditions.

## Conclusion

This study presents a comprehensive characterization of the thermohaline dynamics of the Isla Isabel crater lake, elucidating the physical processes that govern its seasonal heliothermal regime. Our high-resolution, year-long analysis provides novel insights into the energy dynamics of small, meromictic tropical lakes, systems that are highly sensitive to climatic perturbations yet remain underrepresented in the scientific literature.

The results confirm that the lake functions as a heliothermal system where radiative forcing governs the thermal dynamics, while haline stratification, driven by seasonal freshwater inputs, serves as the primary control mechanism. We have demonstrated that the heliothermal condition in this lake is not a persistent, year-round state but a distinct seasonal feature that develops rapidly in response to the rainy season and lasts for approximately eight months before the mixolimnion returns to a well-mixed, isothermal state. This finding challenges any assumption of a persistent state and highlights the system’s strong seasonal variability.

The characterization of this unique environment establishes a critical baseline for understanding the coupling between physical and biogeochemical processes under extreme stratification. Furthermore, the findings of this study offer valuable insights into the dynamics of heat and mass fluxes in the Isla Isabel crater lake. This knowledge is essential for refining models of the physical and climatic processes that govern this unique ecosystem, thereby contributing to the development of more effective strategies for its conservation and sustainable management.

## Data Availability

The datasets used and analysed during the current study available from the corresponding author on reasonable request.
